# Study on OH Radical Production Depending on the Pulse Characteristics in an Atmospheric-Pressure Nanosecond-Pulsed Plasma Jet

**DOI:** 10.3390/ma16103846

**Published:** 2023-05-19

**Authors:** Youbin Seol, Minsu Choi, Hongyoung Chang, Shinjae You

**Affiliations:** 1 Applied Physics Lab for Plasma Engineering (APPLE), Department of Physics, Chungnam National University, Daejeon 34134, Republic of Korea; youbin0621@cnu.ac.kr (Y.S.); bss125576@naver.com (M.C.); 2 Department of Physics, Korea Advanced Institute of Science and Technology, Daejeon 34141, Republic of Korea; hychang@gmail.com

**Keywords:** atmospheric pressure plasma, plasma jet, nanosecond pulse, hydroxyl radical, plasma medicine

## Abstract

Hydroxyl radicals (OH) play a crucial role in plasma-bio applications. As pulsed plasma operation is preferred, and even expanded to the nanosecond range, it is essential to study the relationship between OH radical production and pulse characteristics. In this study, we use optical emission spectroscopy to investigate OH radical production with nanosecond pulse characteristics. The experimental results reveal that longer pulses generate more OH radicals. To confirm the effect of pulse properties on OH radical generation, we conduct computational chemical simulations, focusing on two types of pulse properties: pulse instant power and pulse width. The simulation results show that, similar to the experimental results, longer pulses generate more OH radicals. In the nanosecond range, reaction time is critical for OH radical generation. In terms of chemical aspects, N_2_ metastable species mainly contribute to OH radical generation. It is a unique behavior observed in nanosecond range pulsed operation. Furthermore, humidity can turn over the tendency of OH radical production in nanosecond pulses. In a humid condition, shorter pulses are advantageous for generating OH radicals. Electrons play key roles in this condition and high instant power contributes to them.

## 1. Introduction

Plasma technology is increasingly used across a wide range of fields. Numerous studies have been conducted on plasma physics and plasma applications [1,2,3,4,5,6,7,8]. Atmospheric pressure plasmas, in particular, hold immense potential for both academic research and industrial applications [9,10]. One rapidly growing area is plasma medicine, where atmospheric pressure plasmas are applied to biosystems [11,12]. Research in this field covers a broad range of topics, including sterilization, germination, blood coagulation, and electrosurgery. Plasma medicine has demonstrated significant advantages in treating biosystems [9,11,12,13,14].

Various types of plasma devices have been developed for their bio-applications. Among them, atmospheric pressure plasma jets show their unique characteristics. Needle-shaped, thin, and long discharge can handle localized and concentrated treatment with low damage [9,10,15,16,17,18,19,20].

Normally, a high voltage is required to generate plasmas in atmospheric pressure. In plasma-bio applications, preventing electric shock or heat damage is necessary [12,21]. For a biosystem, a room temperature level under 40 °C is recommended. The pulsed operation of a plasma device can offer both a high performance and a sufficiently low temperature [9,10]. Plasma devices for plasma medicine have recently used even nanosecond range pulses [22]. Pulse signals have many factors to adjust, such as the amplitude of a pulse, the length of a pulse (pulse width), and the repetition rate (frequency) of a pulse. To study a plasma discharge, a pulse signal can be divided into two parts, on-time and off-time. As the excitation power estimation of the plasma is complicated [23], especially complex in a plasma jet with no ground electrode, we adopted the concept of instant power for clear analysis. For the pulse on-time, we can calculate the instant power that equals $\frac{1}{T_{on}}\int_{0}^{T_{on}}V(t)I(t)dt$, which is considerably higher than the total average power [24]. This high instant power mainly contributes to the plasma discharge and its chemistry whereas the instant power of the off-time is considered zero. The instant power is inversely proportional to the pulse duty.

In the interaction between plasma and a biosystem, Reactive Oxygen Species (ROS) are one of the key factors explaining its effect. Among them, the hydroxyl radical (OH) plays an important role in plasma chemistry. Easily generated by plasma under humid conditions with its high oxidative properties, it contributes to the sterilization effect, but deals less damage to the human body [11,25,26,27]. Therefore, the study on the OH production mechanism and its rate is an important issue for plasma-bio applications.

Some reports on OH production in atmospheric pressure pulsed plasma exist, mostly studied in the microsecond pulse range [25,27,28,29,30]. As the voltage and frequency increase, the OH production increases [27] and it fluctuates following the pulse on-time and off-time [28]. As an extension of the works, we studied the OH radical production depending on the pulse characteristics in a nanosecond range.

In this study, we investigate the production of OH radicals in a nanosecond pulsed plasma jet using optical emission spectroscopy. We explore how OH radical production depends on the pulse properties and find that OH production increases with pulse width. To supplement our experimental findings, we adopt chemical simulations to calculate OH production, focusing on pulse width and instant power as pulse parameters. Consistent with our experimental results, we find that OH radical production increases with pulse width. However, we also observe that in humid conditions, the tendency of OH production is reversed.

## 2. Materials and Methods

### 2.1. Optical Emission Spectroscopy

**Figure 1:**
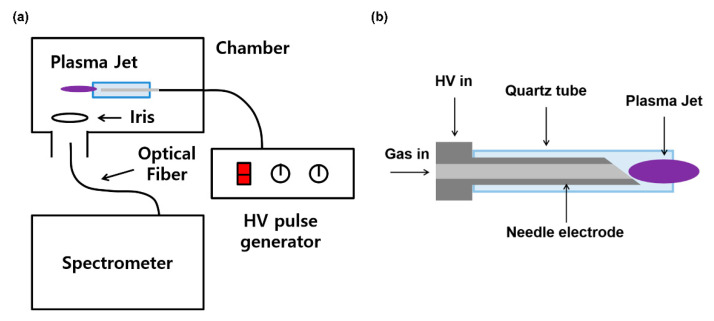
Experimental Settings. (**a**) Schematic design, (**b**) Plasma jet device.

**Figure 2:**
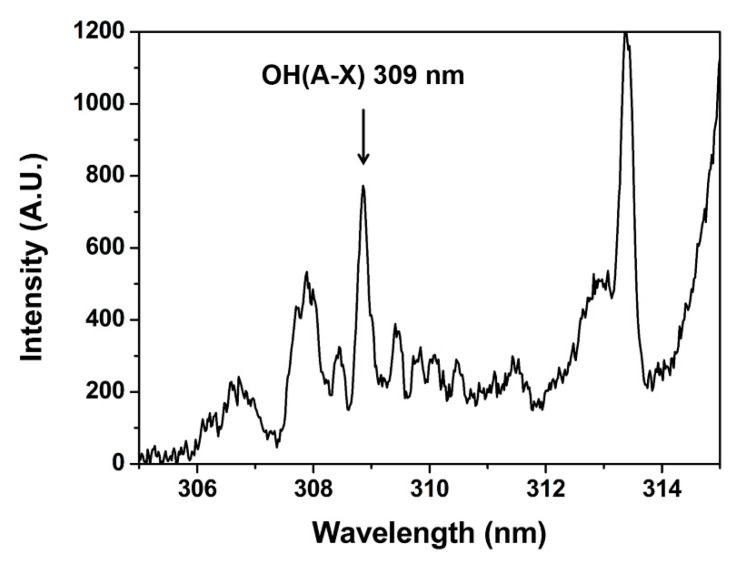
OH emission band.

Figure 1a shows the schematic design of experimental settings. A needle-shaped plasma jet (Figure 1b) is placed in a grounded chamber. The source consists of a 4 cm-long stainless-steel needle and a 4.3 cm-long quartz tube. The inner diameter of the quartz tube is 2 mm, and the outer diameter is 3 mm. Argon gas flows through the needle, and the high-voltage (HV) needle generates a jet plume. A home-made HV nanosecond pulse generator (CNSL, KAIST) was used for the plasma jet operation. It had a peak voltage of 6 kV, two different pulse widths of half-maximums 350 and 700 ns, with a repetition rate of 20 kHz, which gave the same average powers of 1 W with different pulse duties by current control. An OH 309 nm band (Figure 2) was observed by optical emission spectroscopy using a monochromator (DONGWOO OPTRON, Gwangju, Republic of Korea) and an ICCD camera (iStar, Andor, Belfast, Ireland). As the flume length is changed by the input voltage, power and gas flow, iris is used to normalize the emission intensity.

### 2.2. Computational Chemical Simulation

We use a practical chemical simulation tool (Chemkin, Ansys, 2019). The simulation uses a homogeneous 0-D reactor model [31]. The reactor is assumed nearly spatially uniform owing to high diffusion rates. The input power is also deposited uniformly into the plasma bulk. The bulk plasma modeling uses a thin-sheath approximation. The mass balance equation and the energy equation which substitute plasma particle balance equation and energy balance equation for the plasma system and the reaction rate constants govern the simulation [31].

The global mass balance equation can be written as

$$\frac{dm}{dt}=\sum_{}^{N_{inlet}}\dot{m}+\sum_{}^{}{\dot{m}}_{re}-{\dot{m}}_{out}+\sum_{}^{}{\dot{m}}_{surf}$$

which consists of inlet, outlet, recycled and surface mass flow rate terms.

Similarly, the energy equation can be written as

$$\frac{dU}{dt}=\sum_{}^{N_{inlet}}\dot{m}Yh+\sum_{}^{}{\dot{m}}_{re}Yh-{\dot{m}}_{out}Yh-Q_{loss}+Q_{source}-W$$

*Y* is the mass fraction, and *h* is enthalpy. In this case, it additionally includes heat loss, power source and work terms.

The reaction rate constants are considered in Arrhenius form, $k=A{\left(\frac{T}{298K}\right)}^{\beta }\exp\left(\frac{E_{a}}{RT}\right)$. The main reactions considered in the calculation are presented in Table 1.

The inlet gas flow is changed from 1 to 3 slm (standard liter per minute) with a mixed gas of Argon and air, considering the mixing effect in a real atmospheric pressure plasma jet. The total input power is fixed at 1 W and the pulse repetition rate is 10 kHz. We focus on the pulse on-time signals and the estimated instant power is applied to the plasma with the duration of the pulse width. As the lifetime of OH radicals in atmospheric pressure is a few μs, which can be depleted during the off-time of our repetition rate, the peak value at each on-time is used for estimating OH radical production [47,48]. We have changed the pulse widths from 100 ns to 1 μs, which corresponds to pulse duties of 0.1 to 1%, and the instant powers are from 1000 to 100 W, respectively.

## 3. Results and Discussions

### 3.1. Optical Emission Spectroscopy Results

**Figure 3:**
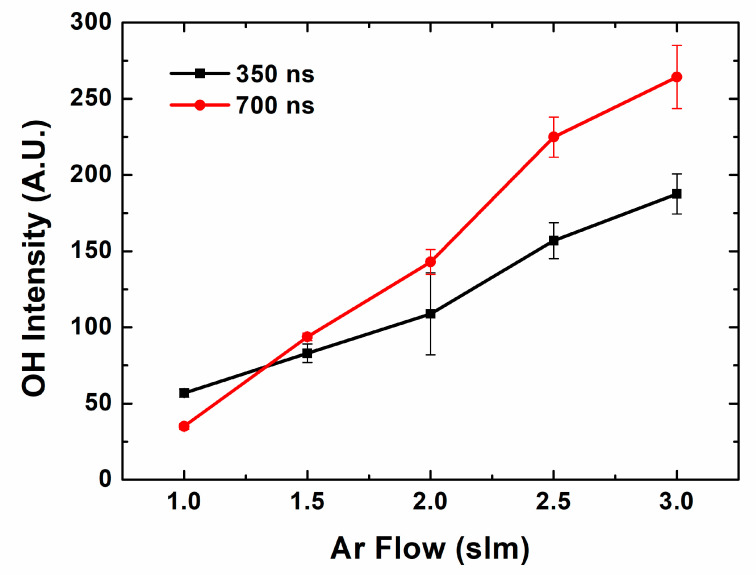
OH intensity on Ar flow in different pulse widths.

In the atmospheric pressure plasma jet, Ar gas is fed and mixed with the air. Hydroxyl radicals are generated from hydrogen, oxygen and water in the air. Figure 3 shows the OH 309 nm band intensity depending on the pulse length. In this case, the pulse width is proportional to the pulse duty, which means it is inversely proportional to the instant power. The OH band intensity can be used to monitor the OH radical density qualitatively [29]. Basically, OH radical generation increases with the gas flow. As Ar gas works as an electron source, plasma discharge efficiency and the electron density increases, which contribute to OH radical generation. Moreover, in this gas flow range, the jet flume expands with the gas flow, which gives a larger discharge volume, and this contributes to OH radical generation. Comparing two pulse cases, in nanosecond pulse range, a longer pulse generally generates more OH radicals. As the gradient of OH radical intensity on Ar gas flow in longer pulse is higher than that of shorter pulse, the difference becomes bigger in higher gas flow. It means that a longer pulse is advantageous for OH radical generation in relatively lower H_2_O conditions. These results are unique characteristics of nanosecond pulsed operation, whereas microsecond pulse show little pulse width effect on the OH radical production [27].

### 3.2. Chemical Simulation Results

**Figure 4:**
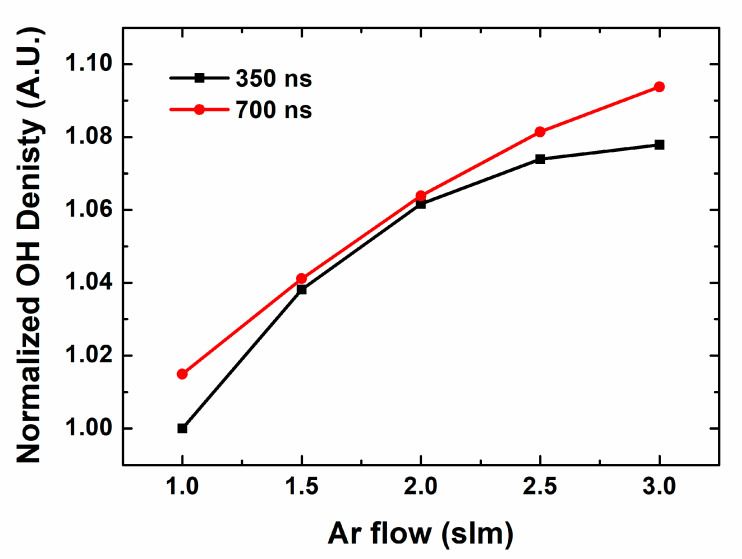
Normalized OH density on Ar flow in different pulse width by computational simulation.

To supplement the experimental results, we have adopted a chemical simulation. OH radical production depending on pulse width is studied in the similar conditions with experimental environment. Figure 4 shows OH radical productions on the Ar gas flow in different pulse widths, 350 and 700 ns. Overall, OH radical productions show similar behaviors to experimental case. It increases with gas flow, and longer pulse shows higher value and gradient. The concrete value of each case shows some difference, with stronger dependence on gas flow in experimental case. In real situation, the size of jet flume is changed by the gas flow, and the fluidal characteristics also affects the shape and emission intensity of the jet flume. Nevertheless, it cannot turn over the trend and correlation of the pulse characteristics, the analysis is still valid.

**Figure 5:**
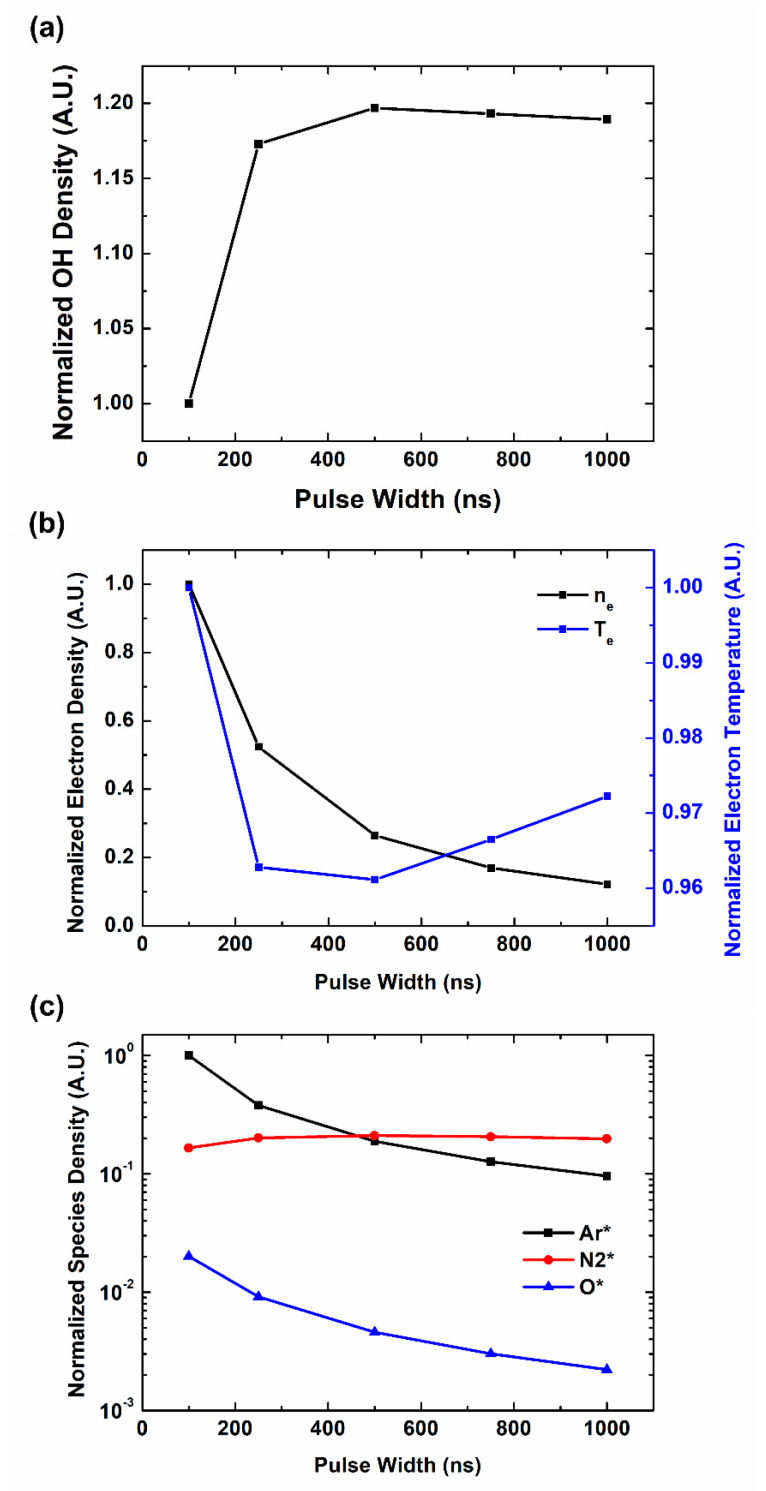
Computational simulation results of plasma species and parameters on pulse width: (**a**) OH density, (**b**) electron parameters, (**c**) reaction participants in plasma.

To study the detailed effect of pulse characteristics on OH radical production, we have expanded the simulation conditions. Figure 5a shows the OH radical density depending on pulse width in different Ar gas flow. Basically, a longer pulse generates more OH radicals, but the effect of the pulse width diminishes as the pulse width reaches the microsecond range. It corresponds to the previous reports on OH radical productions in microsecond pulse range [27].

To verify the detailed contribution of each parameter, we have studied the parameters of several species. These are the main pathways to generate OH radicals in atmospheric plasmas [25,26,49,50,51].

$$e+H_{2}O\to OH+H+e$$

$$N_{2}^{*}+H_{2}O\to OH+N_{2}+H$$

$$O^{*}+H_{2}O\to 2OH$$

The reactions include direct dissociation of H_2_O by electron collision and H_2_O dissociation by metastables. At first, we have focused on the electron parameters. Figure 5b shows the electron parameters on pulse characteristics. Opposite from the OH radical behaviors, electron density and temperature decrease with the pulse width. As the electrons can react to the input power signal fast enough, high instant power contributes to the electron generation, comparing to the pulse width and each pulse energy. The trend of electron density begins to be saturated near μs, where the instant power has become low enough. We also note that each figure shows the peak values of electron parameters in pulse on-time. In real situation, the pulse off time is longer in shorter pulse and this discrepancy can be somewhat compensated. These electron parameter trends do not show a direct relation to OH radical generation. In the nanosecond range, electrons are not the main control factor for OH radical generation. Figure 5c shows the density behaviors of main species which participate in OH radical productions. These species are related to reactions no. 63, 64 and 68. In our results, only N_2_ * distribution shows the relation with OH radical distribution. Similar to OH density behaviors, N_2_ * density increases with the pulse width and it is saturated near microsecond range. It can be interpreted that reaction no. 68 with N_2_ * is the major reaction which controls OH radical distribution.

The increase of OH radical production in longer pulses is due to the length of reaction time. Although the high instant power of the short pulse increases the OH radical production rate, the time to react is extremely short, a few hundred nanoseconds. It cannot meet the saturation point until the pulse on-time ends. However, in longer pulses, enough reaction time is supported which brings production rate closer to the saturation time with moderate power. Again, as the pulse width reaches the microsecond level, instant power becomes lower and the OH radical generation is fully saturated during the pulse on-time.

**Figure 6:**
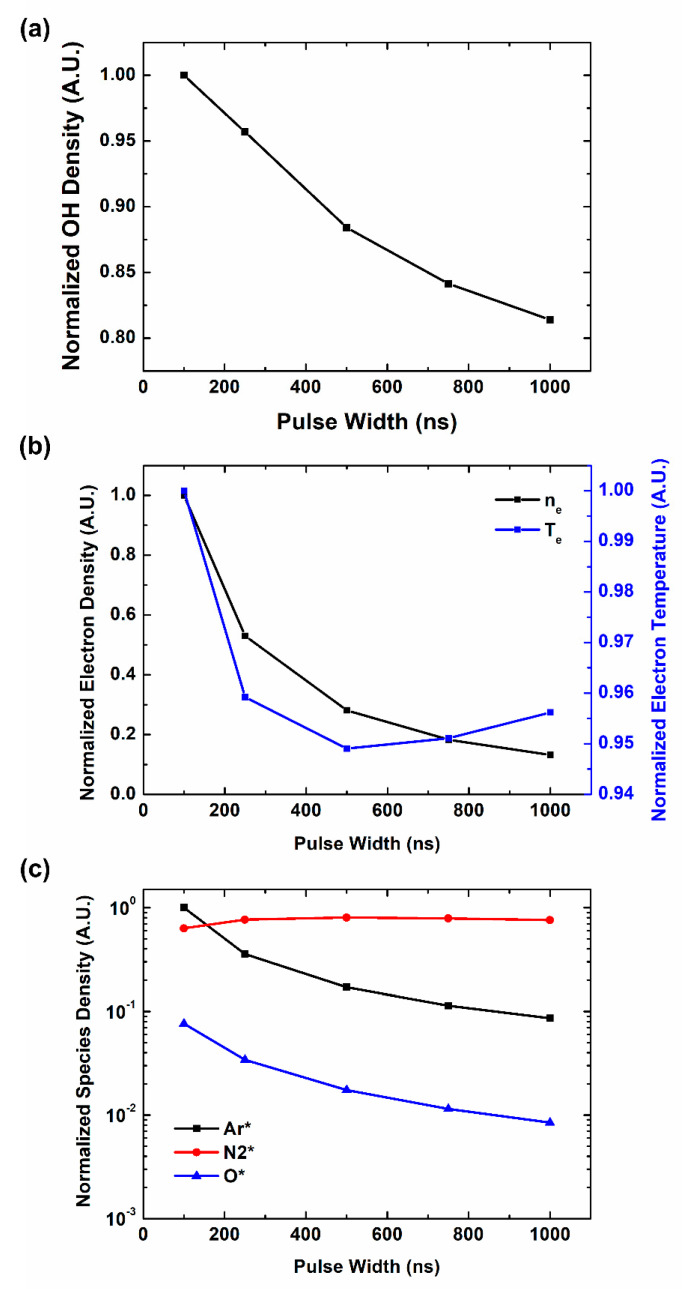
Computational simulation results of plasma species and parameters on pulse width in a humid condition: (**a**) OH density, (**b**) electron parameters, (**c**) reaction participants in plasma.

Humidity is one of the important factors in OH radical production. It is reported that humidity has a significant effect on OH radical production [44,50]. As the source of OH radical in atmospheric pressure is H_2_O, humidity in air can change the main reaction path and the tendency of OH radical production by pulse characteristics. In our works above, the ratio of H_2_O is fixed at 0.2% in the assumption of dry condition. In the case of plasma jet treatment on bio-surfaces, many conditions can be considered to be humid. Therefore, we also study OH radical production by pulse characteristics, using a chemical simulation in a humid condition with H_2_O ratio 2% as shown in Figure 6a. In humid case, OH radical density decreases with pulse width, which is opposite behavior comparing to dry case. Figure 6b shows the behaviors of electron parameters. Both electron density and temperature decrease with the pulse width, which follows the trend of OH radicals. However, behaviors of other reactive species do not show correlation with OH radical productions. Therefore, in humid cases we can find that reactions like no. 61, which accompany electrons, are the main source of OH radical production.

Comparing to dry condition, in humid condition, there exists enough number of H_2_O to collide with electrons, which have very small cross sections. Therefore, direct dissociation of H_2_O by electrons, like reaction no. 72, become the main source of OH radical production [49]. As pulse width increases, the number of electrons and their temperature decreases, OH radical production also decreases. Therefore, in humid conditions, short pulses generate more OH radicals.

## 4. Conclusions

OH radical production in a nanosecond pulsed plasma jet and its dependence on pulse characteristics is studied. Our results demonstrate that pulse width and its instant power contribute to OH radical generation in a complex way. Basically, longer pulses generate more OH radicals due to the increased reaction time. N_2_ metastable is the primary contributor to their distribution comparing to electrons and other species. This result is a unique trait of a plasma operated by a nanosecond pulse.

Additionally, we find that humid conditions can affect OH radical production, with shorter pulses leading to increased OH radical production. In a H_2_O rich condition, high instant power in the shorter pulse contributes to electron parameters and direct dissociation of H_2_O by electrons mainly contribute to OH radical production.

This study verifies the OH radical productions and the electron characteristics in a nanosecond pulsed plasma jet. In academic perspective, this research expanded the understanding of the pulsed plasma jets in a nanosecond range. In practical way, the study will contribute to the optimization of the pulsed plasma jets, especially for the medical uses. In this report, we have focused on the OH radical as chemical species and pulse width as pulse parameters for clear study. For the behaviors of other important species and effect of various pulse parameters, further researches are required.

## Figures and Tables

**Table 1:** Reactions considered in the simulation. The units of reaction rates are in molecules, ${cm}^{n}s^{-1}$, and Kelvin, where n is dependent on reaction order.

No	Reaction	A	β	$E_{a}$	Ref
1	e + Ar → e + Ar *	1.17 × 10^−8^	4.66 × 10^−2^	1.39 × 10^5^	[32]
2	e + Ar → Ar+ + 2e	7.07 × 10^−11^	0.61	1.87 × 10^5^	[32]
3	e + Ar * → Ar+ + 2e	1.25 × 10^−7^	5.04 × 10^−2^	6.05 × 10^4^	[32]
4	e + O_2_ → O + O * + e	4.52 × 10^−13^	0.87	5.11 × 10^4^	[32]
5	e + O_2_ → O_2_+ + 2e	3.99 × 10^−14^	1.13	1.38 × 10^5^	[32,33]
6	e + O_2_ → O + O−	3.60 × 10^−8^	−0.52	5.74 × 10^4^	[32,33]
7	e + O → O * + e	4.30 × 10^−7^	−0.35	3.84 × 10^4^	[32,34]
8	e + O → O+ + 2e	1.95 × 10^−11^	0.62	1.65 × 10^5^	[32,34]
9	e + O * → O+ + 2e	1.95 × 10^−11^	0.62	1.40 × 10^5^	[32]
10	e + O− → O + 2e	2.10 × 10^−10^	0.54	3.94 × 10^4^	[32]
11	e + e + O → O− + e	1.00 × 10^−30^			[32,34]
12	Ar * + Ar * → Ar + Ar+ + e	6.20 × 10^−10^			[32]
13	O− + O_2_+ → O + O_2_	2.80 × 10^−7^			[32]
14	O− + O+ → 2O	2.80 × 10^−7^			[32]
15	O− + O → O_2_ + e	1.40 × 10^−10^			[32]
16	O− + Ar+ → O + Ar	2.80 × 10^−7^			[32]
17	O+ + O_2_ → O_2_+ + O	2.10 × 10^−11^			[32]
18	O_2_+ + Ar → Ar+ + O_2_	5.50 × 10^−11^			[32]
19	Ar+ + O_2_ → Ar + O_2_+	4.60 × 10^−11^			[32]
20	Ar+ + O → O+ + Ar	4.60 × 10^−11^			[32]
21	O * + O_2_ → O + O_2_	3.20 × 10^−11^			[32,35]
22	O * + O → O + O	4.00 × 10^−11^			[32]
23	O * + Ar → Ar + O	4.00 × 10^−11^			[32]
24	O + Ar * → Ar + O	4.00 × 10^−11^			[32]
25	O * + Ar * → O + Ar	4.00 × 10^−11^			[32]
26	O_2_ + Ar * → Ar + O_2_	4.00 × 10^−11^			[32]
27	e + H_2_ → 2H + e	1.70 × 10^−8^	−2.44 × 10^−2^	1.20 × 10^5^	[32]
28	e + H_2_ → H_2_+ + 2e	1.33 × 10^−13^	1.07	1.98 × 10^5^	[32]
29	e + H → H + e	8.37 × 10^−10^	0.30	1.34 × 10^5^	[32]
30	e + H → H+ + 2e	7.33 × 10^−12^	0.69	1.69 × 10^5^	[32]
31	O + O + M = O_2_ + M	5.21 × 10^−35^		−900	[32,36]
32	O + O_3_ = O_2_ + O_2_	8.71 × 10^−12^		2113	[32]
33	O_3_ + M = O + O_2_ + M	7.17 × 10^−10^		1.12 × 10^4^	[32]
34	H_2_ + O_2_ = OH + OH	2.82 × 10^−11^		2.40 × 10^4^	[32,36]
35	OH + H_2_ = H_2_O + H	1.94 × 10^−15^	1.3	1825	[32,36]
36	O + OH = O_2_ + H	6.64 × 10^−10^	−0.5		[32,36]
37	O + H_2_ = OH + H	8.40 × 10^−20^	2.67	3165	[32,36]
38	OH + HO_2_ = H_2_O + O_2_	1.25 × 10^−11^			[32,36]
39	H + HO_2_ = OH + OH	2.33 × 10^−10^		540	[32,36]
40	O + HO_2_ = O_2_ + OH	2.33 × 10^−11^		540	[32,36]
41	OH + OH = O + H_2_O	9.96 × 10^−16^	1.3		[32,36]
42	H + HO_2_ = H_2_ + O_2_	2.08 × 10^−11^			[32,36]
43	HO_2_ + HO_2_ = H_2_O_2_ + O_2_	3.32 × 10^−12^			[32,36]
44	H_2_O_2_ + M = OH + OH + M	2.16 × 10^−7^		2.29 × 10^4^	[32,36]
45	H_2_O_2_ + H = HO_2_ + H_2_	2.66 × 10^−12^		1912	[32,36]
46	H_2_O_2_ + OH = H_2_O + HO_2_	1.66 × 10^−11^		906	[32,36]
47	H + O_2_ + M = HO_2_ + M	9.95 × 10^−31^	−0.72		[32,36]
48	H + H + M = H_2_ + M	2.76 × 10^−30^	−1		[32,36]
49	H + H + H_2_ = H_2_ + H2	2.54 × 10^−31^	−0.6		[32,36]
50	H + H + H_2_O = H_2_ + H_2_O	1.65 × 10^−28^	−1.25		[32,36]
51	H + OH + M = H_2_O + M	4.41 × 10^−26^	−2		[32,36]
52	H + O + M = OH + M	1.71 × 10^−31^	−0.6		[32,36]
53	H_2_+ + Ar → Ar+ + H_2_	3.00 × 10^−10^			[32,37]
54	Ar+ + H_2_ → H_2_+ + Ar	1.30 × 10^−10^			[32,37]
55	e + N_2_ → e + N_2_ *	5.65 × 10^−21^	2.17	2.90 × 10^4^	[38,39]
56	e + N_2_ → N_2_+ + 2e	2.56 × 10^−43^	7.07	3.15 × 10^4^	[38,39]
57	e + N → N+ + 2e	5.11 × 10^−37^	5.78	4.76 × 10^4^	[38,39]
58	e + N+ → N	2.25 × 10^−1^	−2.5		[38,39]
59	e + N_2_+ → N + N	2.25 × 10^−1^	−2.5		[38,39]
60	e + N_2_+ → N_2_	2.25 × 10^−1^	−2.5		[38,39]
61	e + H_2_O → OH + H + e	1.37 × 10^−6^	−0.34	1.63 × 10^5^	[40]
62	e + H_2_O → OH * + H + e	4.04 × 10^−3^	−1.13	2.07 × 10^5^	[40]
63	O * + H_2_O → 2OH	2.20 × 10^−10^			[35]
64	Ar * + H_2_O → OH + H + Ar	4.50 × 10^−10^			[41]
65	Ar * + H_2_O → Ar + H_2_O+ + e	4.50 × 10^−10^			[42]
66	Ar+ + H_2_O → H+ + OH + Ar	3.10 × 10^−10^			[40]
67	e + H_2_O+ → OH + H	2.60 × 10^−8^			[42]
68	N_2_ * + H_2_O → OH + N_2_ + H	5.00 × 10^−14^			[43]
69	N_2_ * + OH → OH * + N_2_	1.00 × 10^−10^			[43]
70	e + H_2_O_2_ → 2OH + e	2.36 × 10^−9^			[40]
71	e + OH → e + OH *	2.70 × 10^−10^			[44]
72	e + H_2_O → H_2_O+ + 2e	3.08 × 10^−20^	2.33	1.31 × 10^5^	[40]
73	OH * → OH	1.25 × 10^12^			[40]
74	NO + O → O_2_ + N	8.93 × 10^−13^	1	1.62 × 10^5^	[45]
75	NO + N → N_2_ + O	3.11 × 10^−11^			[35]
76	NO → O + N	1.60 × 10^−9^		6.20 × 10^5^	[45]
77	O * + N_2_ → N_2_ + O	1.79 × 10^−11^		−890	[35]
78	O_3_ + N → O_2_ + NO	1.00 × 10^−16^			[35]
79	O_2_ + N → NO + O	4.47 × 10^−12^	1	2.72 × 10^4^	[46]
80	OH + N → NO + H	3.80 × 10^−11^		−707	[35]

## Data Availability

No new data were created or analyzed in this study. Data sharing is not applicable to this article.

## References

[1] Conrads H., Schmidt M. (2000). Plasma Generation and Plasma Sources. Plasma Sources Sci. Technol..

[2] Chapman B. (1980). Glow Discharge Processes: Sputtering and Plasma Etching.

[3] Lieberman M.A., Lichtenberg A.J. (2005). Principles of Plasma Discharges and Materials.

[4] Kushner M.J. (1982). A Kinetic Study of the Plasma-Etching Process. II. Probe Measurements of Electron Properties in an Rf Plasma-Etching Reactor. J. Appl. Phys..

[5] Godyak V.A., Piejak R.B. (1990). Abnormally Low Electron Energy and Heating-Mode Transition in a Low-Pressure Argon Rf Discharge at 13.56 MHz. Phys. Rev. Lett..

[6] Vender D., Boswell R.W. (1990). Numerical Modeling of Low-Pressure RF Plasmas. IEEE Trans. Plasma Sci..

[7] Lee J.J., Kim S.J., Lee Y.S., Cho C.H., Choi M.S., Seong I.H., Lee S.H., Jeong W.N., You S.J. (2020). Experimental Study of Argon and Helium Dielectric Barrier Discharge with Coplanar Electrodes at Intermediate Pressure for Reducing Radar Cross Section. Appl. Sci. Converg. Technol..

[8] Seong I.H., Lee J.J., Cho C.H., Lee Y.S., Kim S.J., You S.J. (2021). Characterization of SiO2 Over Poly-Si Mask Etching in Ar/C4F8 Capacitively Coupled Plasma. Appl. Sci. Converg. Technol..

[9] Tendero C., Tixier C., Tristant P., Desmaison J., Leprince P. (2006). Atmospheric Pressure Plasmas: A Review. Spectrochim. Acta Part B At. Spectrosc..

[10] Nehra V., Kumar A., Dwivedi H.K. (2008). Atmospheric Non-Thermal Plasma Sources. Int. J. Eng..

[11] Dobrynin D., Fridman G., Friedman G., Fridman A. (2009). Physical and Biological Mechanisms of Direct Plasma Interaction with Living Tissue. New J. Phys..

[12] Lloyd G., Friedman G., Jafri S., Schultz G., Fridman A., Harding K. (2010). Gas Plasma: Medical Uses and Developments in Wound Care. Plasma Process. Polym..

[13] Fridman G., Peddinghaus M., Ayan H., Fridman A., Balasubramanian M., Gutsol A., Brooks A., Friedman G. (2006). Blood Coagulation and Living Tissue Sterilization by Floating-Electrode Dielectric Barrier Discharge in Air. Plasma Chem. Plasma Process..

[14] Ling L., Jiafeng J., Jiangang L., Minchong S., Xin H., Hanliang S., Yuanhua D. (2014). Effects of Cold Plasma Treatment on Seed Germination and Seedling Growth of Soybean. Sci. Rep..

[15] Schutze A., Jeong J.Y., Babayan S.E., Park J., Selwyn G.S., Hicks R.F. (1998). The Atmospheric-Pressure Plasma Jet: A Review and Comparison to Other Plasma Sources. Plasma Sci. IEEE Trans..

[16] Lu X., Laroussi M., Puech V. (2012). On Atmospheric-Pressure Non-Equilibrium Plasma Jets and Plasma Bullets. Plasma Sources Sci. Technol..

[17] Jiang N., Ji A., Cao Z. (2009). Atmospheric Pressure Plasma Jet: Effect of Electrode Configuration, Discharge Behavior, and Its Formation Mechanism. J. Appl. Phys..

[18] Shrestha R., Gurung J.P., Subedi D.P., Wong C.S. (2015). Atmospheric Pressure Single Electrode Argon Plasma Jet for Biomedical Applications. Int. J. Emerg. Technol. Adv. Eng..

[19] Lu X., Jiang Z., Xiong Q., Tang Z., Pan Y. (2008). A Single Electrode Room-Temperature Plasma Jet Device for Biomedical Applications. Appl. Phys. Lett..

[20] Zhang X., Liu D., Zhou R., Song Y., Sun Y., Zhang Q., Niu J., Fan H., Yang S. (2014). Atmospheric Cold Plasma Jet for Plant Disease Treatment. Appl. Phys. Lett..

[21] Baik K.Y., Kang H.L., Kim J., Park S.Y., Bang J.Y., Uhm H.S., Choi E.H., Cho G. (2013). Non-Thermal Plasma Jet without Electrical Shock for Biomedical Applications. Appl. Phys. Lett..

[22] Boselli M., Colombo V., Gherardi M., Laurita R., Liguori A., Sanibondi P., Simoncelli E., Stancampiano A. (2015). Characterization of a Cold Atmospheric Pressure Plasma Jet Device Driven by Nanosecond Voltage Pulses. IEEE Trans. Plasma Sci..

[23] Lomaev M.I. (2001). Determination of Energy Input in Barrier Discharge Excilamps. Atmos. Ocean. Opt..

[24] Wen F., Lo Y.-L., Lin C.-H., Mou S.-C. (2006). A Pulse DC Plasma Deposited Resistor Process. Mater. Sci. Forum.

[25] Kanazawa S., Kawano H., Watanabe S., Furuki T., Akamine S., Ichiki R., Ohkubo T., Kocik M., Mizeraczyk J. (2011). Observation of OH Radicals Produced by Pulsed Discharges on the Surface of a Liquid. Plasma Sources Sci. Technol..

[26] Pei X., Lu Y., Wu S., Xiong Q., Lu X. (2013). A Study on the Temporally and Spatially Resolved OH Radical Distribution of a Room-Temperature Atmospheric-Pressure Plasma Jet by Laser-Induced Fluorescence Imaging. Plasma Sources Sci. Technol..

[27] Pei X., Wu S., Xian Y., Lu X., Pan Y. (2014). On OH Density of an Atmospheric Pressure Plasma Jet by Laser-Induced Fluorescence. IEEE Trans. Plasma Sci..

[28] Liu X.Y., Pei X.K., Ostrikov K., Lu X.P., Liu D.W. (2014). The Production Mechanisms of OH Radicals in a Pulsed Direct Current Plasma Jet. Phys. Plasmas.

[29] Shin D.N., Park C.W., Hahn J.W. (2000). Detection of OH and O Emission Spectrum Generated in a Pulsed Corona Plasma. Bull. Korean Chem. Soc..

[30] Ono R., Nakagawa Y., Oda T. (2011). Effect of Pulse Width on the Production of Radicals and Excited Species in a Pulsed Positive Corona Discharge. J. Phys. D Appl. Phys..

[31] K.W.TECH Co., Ltd. (2013). Reaction Design CHEMKIN Theory Manual.

[32] Meeks E., Larson R.S., Ho P., Apblett C., Han S.M., Edelberg E., Aydil E.S. (1998). Modeling of SiO_2_ Deposition in High Density Plasma Reactors and Comparisons of Model Predictions with Experimental Measurements. J. Vac. Sci. Technol. A Vac. Surf. Film..

[33] Itikawa Y., Ichimura A., Onda K., Sakimoto K., Takayanagi K. (1989). Cross Sections for Collisions of Electrons and Photons with Oxygen Molecules. J. Phys. Chem. Ref. Data.

[34] Itikawa Y., Ichimura A. (1990). Cross Sections for Collisions of Electrons and Photons with Atomic Oxygen. J. Phys. Chem. Ref. Data.

[35] Atkinson R., Baulch D.L., Cox R.A., Hampson R.F., Kerr J.A., Troe J. (1989). Evaluated Kinetic and Photochemical Data for Atmospheric Chemistry: Supplement IV. IUPAC Subcommittee on Gas Kinetic Data Evaluation for Atmospheric Chemistry. J. Phys. Chem. Ref. Data.

[36] Miller J.A., Melius C.F. (1992). Kinetic and Thermodynamic Issues in the Formation of Aromatic Compounds in Flames of Aliphatic Fuels. Combust. Flame.

[37] Phelps A.V. (1992). Collisions of H+, H2+, H3+, ArH+, H−, H, and H2 with Ar and of Ar+ and ArH+ with H2 for Energies from 0.1 EV to 10 KeV. J. Phys. Chem. Ref. Data.

[38] Meeks E., Larson R.S., Vosen S.R., Shon J.W. (1997). Modeling Chemical Downstream Etch Systems for NF3/O_2_ Mixtures. J. Electrochem. Soc..

[39] Gangoli S.P. (2007). Experimental and Modeling Study of Warm Plasmas and Their Applications. PhD Thesis.

[40] Tavant A., Lieberman M.A. (2016). Hybrid Global Model of Water Cluster Ions in Atmospheric Pressure Ar/H_2_O RF Capacitive Discharges. J. Phys. D. Appl. Phys..

[41] Bruggeman P., Schram D.C. (2010). On OH Production in Water Containing Atmospheric Pressure Plasmas. Plasma Sources Sci. Technol..

[42] Ghimire B., Sornsakdanuphap J., Hong Y.J., Uhm H.S., Weltmann K.D., Choi E.H. (2017). The Effect of the Gap Distance between an Atmospheric-Pressure Plasma Jet Nozzle and Liquid Surface on OH and N2 Species Concentrations. Phys. Plasmas.

[43] Herron J.T. (1999). Evaluated Chemical Kinetics Data for Reactions of N(2D), N(2P), and N2(A3Σ+u) in the Gas Phase. J. Phys. Chem. Ref. Data.

[44] Li L., Nikiforov A., Xiong Q., Britun N., Snyders R., Lu X., Leys C. (2013). OH Radicals Distribution in an Ar-H_2_O Atmospheric Plasma Jet. Phys. Plasmas.

[45] Tsang W., Herron J.T. (1991). Chemical Kinetic Data Base for Propellant Combustion I. Reactions Involving NO, NO2, HNO, HNO2, HCN and N2O. J. Phys. Chem. Ref. Data.

[46] Baulch D.L., Cobos C.J., Cox R.A., Frank P., Hayman G., Just T., Kerr J.A., Murrells T., Pilling M.J., Troe J. (1994). Evaluated Kinetic Data for Combustion Modelling. Supplement I. J. Phys. Chem. Ref. Data.

[47] Komuro A., Ono R., Oda T. (2013). Behaviour of OH Radicals in an Atmospheric-Pressure Streamer Discharge Studied by Two-Dimensional Numerical Simulation. J. Phys. D Appl. Phys..

[48] Attri P., Kim Y.H., Park D.H., Park J.H., Hong Y.J., Uhm H.S., Kim K., Fridman A., Choi E.H. (2015). Generation Mechanism of Hydroxyl Radical Species and Its Lifetime Prediction during the Plasma-Initiated Ultraviolet (UV) Photolysis. Sci. Rep..

[49] Sun B., Liu D., Iza F., Wang S., Yang A., Liu Z., Rong M., Wang X. (2019). Global Model of an Atmospheric-Pressure Capacitive Discharge in Helium with Air Impurities from 100 to 10,000 Ppm. Plasma Sources Sci. Technol..

[50] Yue Y., Wu F., Cheng H., Xian Y., Liu D., Lu X., Pei X. (2017). A Donut-Shape Distribution of OH Radicals in Atmospheric Pressure Plasma Jets. J. Appl. Phys..

[51] Vasko C.A., Liu D.X., van Veldhuizen E.M., Iza F., Bruggeman P.J. (2014). Hydrogen Peroxide Production in an Atmospheric Pressure RF Glow Discharge: Comparison of Models and Experiments. Plasma Chem. Plasma Process..

